# Psammomatoid juvenile ossifying fibroma of frontal sinus – surgical and reconstructive approach

**DOI:** 10.4322/acr.2021.411

**Published:** 2022-11-30

**Authors:** Jefferson Rocha Tenório, Paulo Roberto de Alencar Bártholo, Mário José Romañach, Aline Corrêa Abrahão, Michelle Agostini, Bruno Augusto Benevenuto de Andrade

**Affiliations:** 1 Universidade Federal do Rio de Janeiro (UFRJ), Faculdade de Odontologia, Departamento de Patologia e Diagnóstico Oral, Rio de Janeiro, RJ, Brasil; 2 Cirurgia e Traumatologia Bucomaxilofacial, Clínica Particular, Manaus, AM, Brasil

**Keywords:** Fibroma, Ossifying, Neoplasms, Bone Tissue, Reconstructive Surgical Procedures, rehabilitation

## Abstract

Psammomatoid juvenile ossifying fibroma (PJOF) is a benign fibro-osseous lesion that mainly affects the paranasal sinuses and periorbital bones. It may cause significant esthetic and functional impairment. Herein, we describe the diagnosis and surgical approach of an extensive PJOF arising in the frontal sinus of a young male. After complete lesion removal and histopathological confirmation, the bone defect was repaired with a customized polymethylmethacrylate implant. PJOF may present aggressive clinical behavior. The excision of extensive PJOF in the orbitofrontal area can result in significant esthetic defects. Polymethacrylate implants restore functionally and esthetically the involved area.

## INTRODUCTION

Juvenile ossifying fibroma (JOF) is a benign fibro-osseous lesion. It is considered a distinct condition from the cemento-ossifying fibroma (COF), because of its non-odontogenic origin and particular clinicopathological features.^1^^,^ 2 In addition, JOF presents a peculiar clinical behavior, with two clinicopathological variants: trabecular JOF (TJOF) and psammomatoid JOF (PJOF).^1^^,^ 2

While TJOF is more common in the maxilla of individuals up to 12 years of age, available clinical data demonstrate that PJOF can occur not only in young people but in individuals ranging from 3 months to 72 years old.^3^ PJOF have no sex predominance and mainly affects extragnathic sites, especially the paranasal sinuses, periorbital bones, and skull base.^3^ Microscopically, the TJOF shows a mineralized component composed of highly cellular osteoid, rich in osteoblastic paving, bulky osteoblasts and multinucleated osteoclasts.^1^^,^ 2 The term psammomatoid, which is used in the PJOF, refers to the calcified, lamellar, concentric, acellular, and basophilic structures commonly observed microscopically.^4^ These structures resemble the psammoma bodies found in papillary thyroid carcinoma, meningioma, and other neoplastic conditions.^5^^,^ 6

Clinically, PJOF of the paranasal sinuses may present ocular proptosis, vision impairment, headaches, nasal congestion, recurrent sinusitis, and marked facial asymmetry.^7^^,^ 8 The excision of the PJOF is usually facilitated by its well-defined appearance; however, the removal of extensive lesions can result in a remarkable esthetic defect, which requires complex rehabilitation and reconstructive techniques.^9^

Herein we report the diagnosis and surgical approach of an extensive PJOF arising from the frontal sinus of a young man, highlighting aspects related to reconstruction through prototyping.

## CASE REPORT

**Figure 1:**
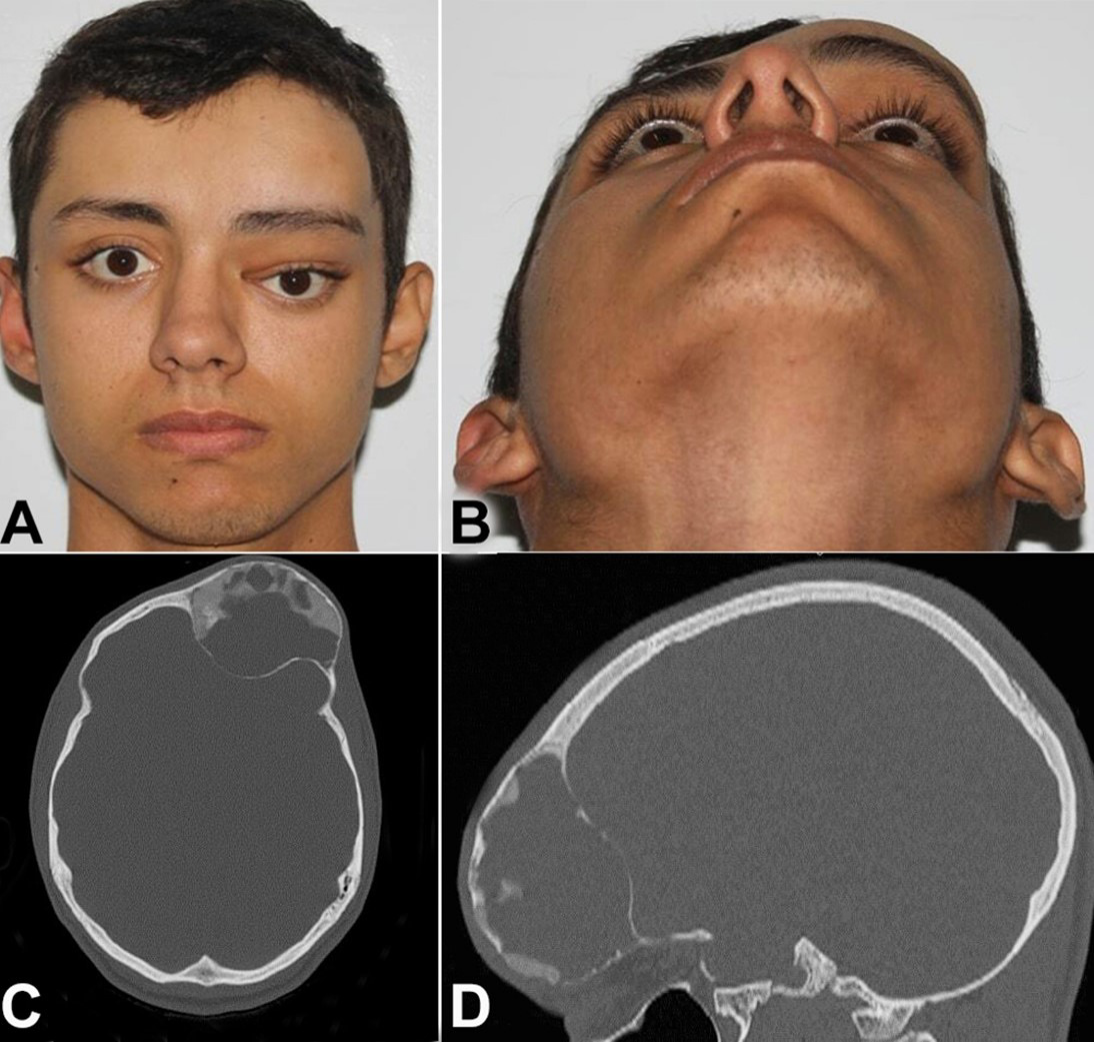
Clinical and imaging aspects. **A –** front view showing ocular dystopia; **B –** infero-superior view, highlighting the expansive aspect of the lesion; **C –** CT axial section, showing a mixed unilocular lesion; **D –** CT sagittal section, showing the preservation of the anterior and posterior cortices.

A 20-year-old white male was referred to the oral and maxillofacial surgery service, complaining of painless facial swelling. He reported the lesion appeared approximately 10 years ago after trauma, with slow progression, but associated with significant esthetic discomfort. His medical history was non-contributory. The physical examination showed a noticeable swelling in the orbitofrontal region of the left side, covered by normal skin. It was firm on palpation (Figures 1A, B). In addition, there was a severe degree of ocular dystopia but no loss of visual acuity and ocular motility. The computed tomography showed a multilocular well-defined, predominantly hypodense, expanding lesion located in the left side of the frontal sinus, with a consequent increase in the ipsilateral orbital cone (Figure 1C and 1D). Based on these characteristics, the patient was submitted to the lesion excisional biopsy under general anesthesia.

**Figure 2:**
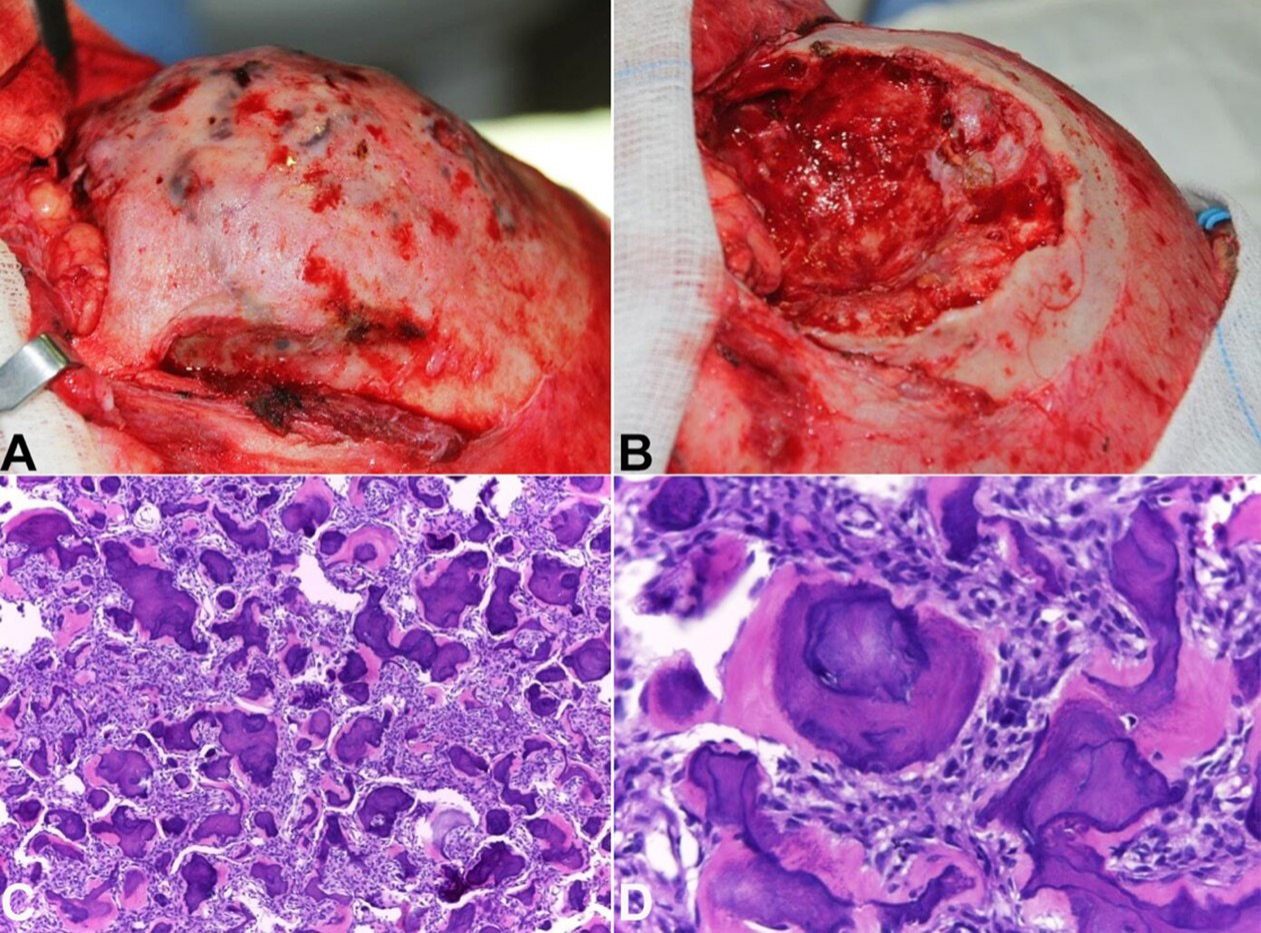
Surgical and histopathological aspects. **A –** Coronal surgical access; **B –** Surgical site after curettage and peripheral osteotomy; **C –** Histopathological examination (HE, 100X) showing a hypercellular stroma and multiple intermingled calcified structures; **D –** Histopathological examination (HE, 200X) emphasizing the psammomatoid bodies.

Coronal access and osteotomy of the anterior wall of the frontal bone were performed for adequate lesion exposure (Figure 2A). As the lesion was well-circumscribed with a clear cleavage plane, it was possible to perform complete excision through curettage and peripheral osteotomy (Figure 2B).

The collected specimen was submitted to histological analysis, which showed a cellular stroma, without mitotic activity or cellular atypia, in addition to multiple rounded calcified structures (Figure 2C). These structures were concentric, basophilic, and lamellar with eosinophilic rimming and were found throughout the lesion (Figure 2D). Based on the clinicopathological and imaging findings, the diagnosis of PJOF was made.

**Figure 3:**
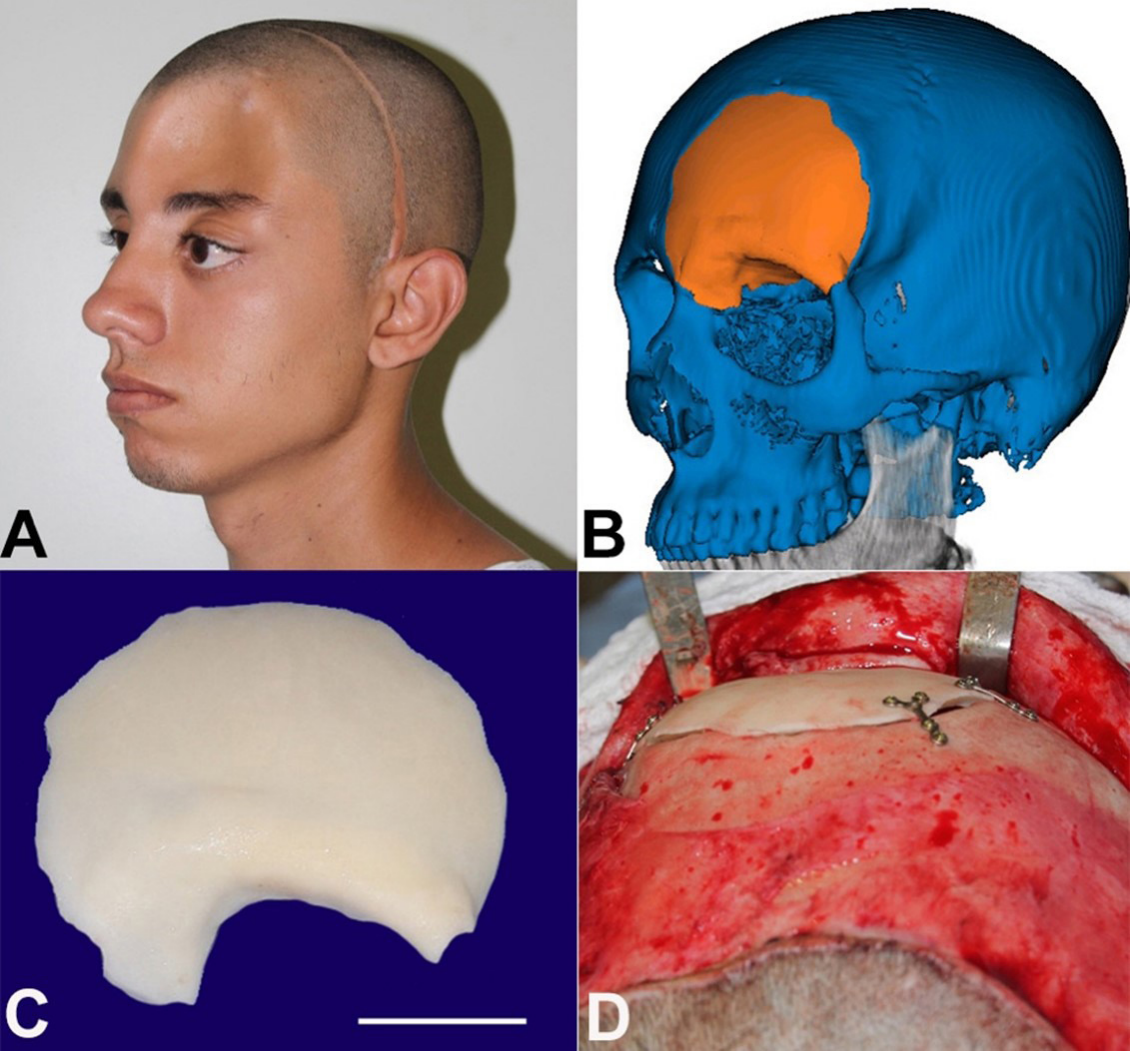
Planning and rehabilitation. **A –** Postoperative appearance after the first surgical intervention showing concavity with a noticeable aesthetic defect; **B –** Planning guided by CT; **C –** Polymethylmethacrylate implant (scale bar = 2,5 cm); **D –** Implant fixed with plates and screws from the fixation system1.5.

As the lesion removal resulted in a concave unfavorable aesthetic defect (Figure 3A), a reconstructing rehabilitation plan was carried out through 3D prototyping (Figure 3B). Thus, guided by the CT image, a customized polymethylmethacrylate implant was performed (Figure 3C). Under general anesthesia, new coronal access was performed, and careful dissection of adjacent structures was performed. The implant was positioned and fixed in the surgical site with several titanium plates and screws from the 1.5 fixation system (Figure 3D).

**Figure 4:**
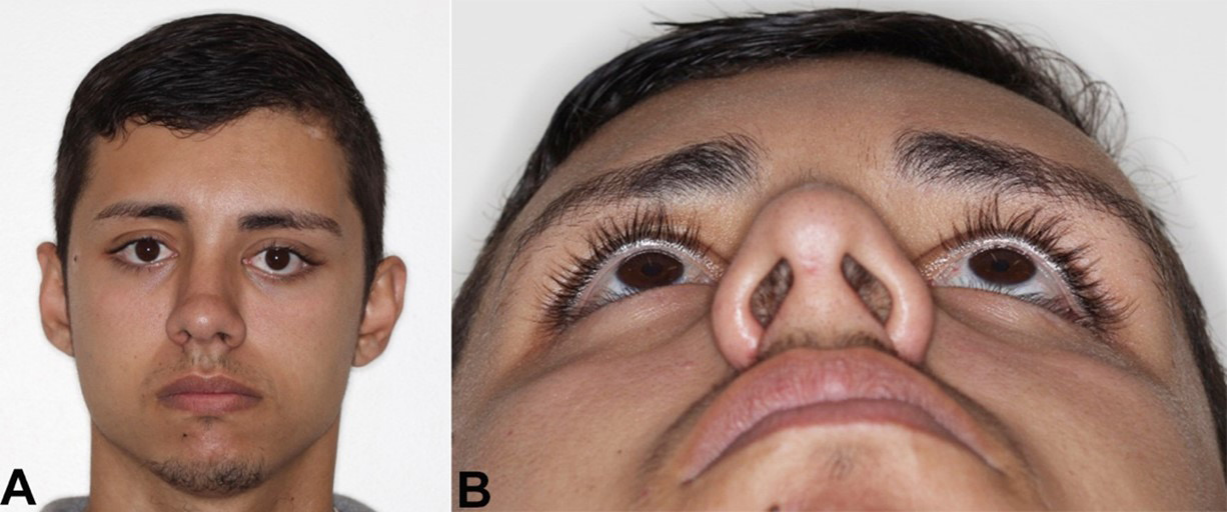
Final postoperative period (06 months). **A –** Frontal view with significant improvement in aesthetic appearance, but with slight remaining ocular dystopia; **B –** Inferior-superior view.

The initial postoperative period showed significant aesthetic improvement (Figures 4A, B), with a slight degree of remaining ocular dystopia. After 06 years of performing the reconstructive procedure, the patient reported no changes in his face or visual function.

## DISCUSSION

This report presents a clinical case of PJOF, which despite being asymptomatic, the lesion caused marked aesthetic deformity, requiring extensive rehabilitation treatment. Bone expansion, mixed radiodensity, and absence of cortical perforation are frequently reported in both JOF variants^2^ and were observed in the present case. Most of JOFs occur in young individuals.

The case reported herein is a PJOF affecting a 20-year-old young man; however, there is wide variability in the age of involvement of the JOF of both variants. Thus, the term “juvenile” is probably inappropriate.^3^^,^ 10

Craniofacial fibrous dysplasia (CFD) is the main differential diagnosis and can mimic a PJOF. CFD is a developmental condition that can affect the patient’s maxillary, zygomatic, temporal, sphenoid, and frontal bones in the second and third decades of life. CFD is rarer than PJOF; its radiographic appearance shows opacification of the bone in a "ground glass" appearance and a lack of distinction between the affected and healthy bones.^11^ Since fibro-osseous lesions have different clinical behavior and treatments; in the present report, the authors emphasized the need for an in-depth clinical history, imaging tests, and histopathological evaluation for making a precise diagnosis.

In addition to the aforementioned differences between PJOF and TJOF, some clinical characteristics seem to be similar.^1^^,^ 2 A recent systematic review showed that there are no statistically significant differences between the JOF variants in the following aspects: sex distribution, the prevalence of bone expansion, pain, cortical bone perforation, the appearance of locularity on radiological examination, radiodensity, radiological limits, cortical bone perforation, presence of a secondary aneurysmal bone cyst, tooth displacement, dental root resorption, and recurrence rate.^3^ However, more than an academic concern, in the present case, this distinction was necessary since the PJOFs frequently involve the sinonasal region and seem to have higher recurrence rates than the trabecular variant.

Several treatments have been proposed for PJOF. A recent systematic review showed that, regardless of the anatomical site, only enucleation or enucleation plus curettage had a high recurrence rate.^3^ On the other hand, recurrence rates were lower when enucleation was associated with peripheral osteotomy.^3^ In this report, considering the trans-surgical aspect of the lesion, which was easily cleaved, the therapeutic approach was based not only on the total lesion excision but also on curettage and peripheral osteotomy, as currently recommended.^3^

Challenges in reconstructing the frontal and orbital regions were faced due to the extent of our case’s lesion. The lesion’s proximity to noble structures, such as the eyeball and the anterior portion of the brain, emerged concern that surgical procedures could lead to visual disturbances, poor eye positioning, partial loss of brain protection, and esthetic defects that could menace the patient´s self-esteem.^9^ These concerns require the oral and maxillofacial surgeon to have knowledge of advanced reconstructive techniques guided by 3D technology. In our case, the reconstructive procedure resulted in favorable esthetics without damaging the surrounding structures.

Different materials can be used to reconstruct lost cranial areas, with their indications and disadvantages.^12^ The most used materials are polyetheretherketone, titanium mesh, methyl methacrylate, hydroxyapatite, and alumina ceramics. We chose the polymethylmethacrylate implant because of its satisfactory hardness, strength, non-irritating and non-conductive.^12^^,^ 13 However, some studies report a high rate of infection associated with this implant compared to other types.^13^^,^ 14 Even though we chose to use this material because of its availability and the surgeon's experience. No short-term and long-term postoperative complications associated with polymethylmethacrylate implants were observed.

## CONCLUSION

PJOFs in the orbitofrontal region may present an aggressive clinical behavior and require specific reconstructive techniques. In the present case, the total removal of the lesion, with curettage, peripheral osteotomy and reconstruction with polymethacrylate implants, proved to be a suitable surgical-reconstructive approach.
